# Auditory-cognitive training for adult cochlear implant recipients: a study protocol for a randomised controlled trial

**DOI:** 10.1186/s13063-021-05714-7

**Published:** 2021-11-12

**Authors:** Blake J. Lawrence, Robert H. Eikelboom, Dona M. P. Jayakody

**Affiliations:** 1 School of Population Health, Curtin University, Bentley, WA Australia; 2 Ear Science Institute Australia, 1 Salvado Road, Subiaco, WA 6008 Australia; 3 Ear Sciences Centre, Medical School, The University of Western Australia, Crawley, WA Australia; 4 Department of Speech-Language Pathology and Audiology, University of Pretoria, Pretoria, South Africa

**Keywords:** Cochlear implant, Auditory-cognitive training, Hearing loss, Cognitive decline, Cognitive function, Auditory rehabilitation

## Abstract

**Background:**

There is an urgent need to develop new therapies to improve cognitive function in adults following cochlear implant surgery. This study aims to determine if completing at-home computer-based brain training activities improve memory and thinking skills in adults following their first cochlear implant.

**Methods:**

This study will be conducted as a single-blind, head-to-head, randomised controlled trial (RCT). It will determine whether auditory training combined with adaptive computerised cognitive training will elicit greater improvement in cognition, sound and speech perception, mood, and quality of life outcomes in adult cochlear implant recipients, when compared to auditory training combined with non-adaptive (i.e. placebo) computerised cognitive training. Participants 18 years or older who meet the clinical criteria for a cochlear implant will be recruited into the study.

**Results:**

The results of this trial will clarify whether the auditory training combined with cognitive training will improve cognition, sound and speech perception, mood, and quality of life outcomes in adult cochlear implant recipients.

**Discussion:**

We anticipate that our findings will have implications for clinical practice in the treatment of adult cochlear implant recipients.

**Trial registration:**

Australian New Zealand Clinical Trials Registry ACTRN12619000609156. Registered on April 23 2019.

## Background

Approximately 2% of the world’s population live with severe to profound hearing loss, based on the prevalence in the USA; this estimate is expected to rise with the world’s ageing population [1, 2]. Hearing loss is associated with cognitive decline [3] and has been identified as the largest contributing, but modifiable, mid-life risk factor of dementia [4]. A cochlear implant combined with post-operative auditory rehabilitation (i.e. auditory training) is now a common and safe treatment for restoring speech and sound perception in people with severe to profound hearing loss [5, 6], whereas the benefits of a cochlear implant combined with auditory training for improving impaired cognitive function (potentially) associated hearing loss are less clear.

A recent systematic review evaluated studies reporting the effects of cochlear implantation on cognitive function in adults [7]. Among the six included, five studies reported improved cognition following cochlear implantation. However, all these studies were limited by methodological biases that undermined reliable interpretation of results [8–13]. Moreover, only two studies reported that participants completed post-operative auditory rehabilitation [8, 12], despite clinical guidelines recommending auditory rehabilitation for successful use and adjustment to a new cochlear implant [14]. It is therefore not clear whether cochlear implantation or cochlear implantation combined with post-operative auditory rehabilitation has the potential to improve cognition in adults following surgery.

Other non-invasive techniques, such as computerised cognitive training (CCT), may complement existing auditory rehabilitation by improving specific domains of cognition associated with speech and sound perception in adult cochlear implant recipients. CCT refers to engagement with standardised, cognitively challenging tasks, to improve cognitive function [15], whereas auditory training refers to active engagement with sounds, where participants learn to make distinctions between sounds presented systematically [16]. Evidence suggests that combining CCT with auditory training may elicit optimal neural conditions to improve cognition in adults with hearing loss [17]. Cochlear implant recipients may therefore experience cognitive benefits from an integrated auditory + CCT intervention as part of their initial rehabilitation following surgery.

The current study will therefore examine whether combining auditory training with adaptive CCT will provide greater improvement in cognition, sound/speech perception, mood, and quality of life in adult cochlear implant recipients, when compared to auditory training combined with non-adaptive (i.e. placebo) CCT.

## Methods/design

**Fig. 1:**
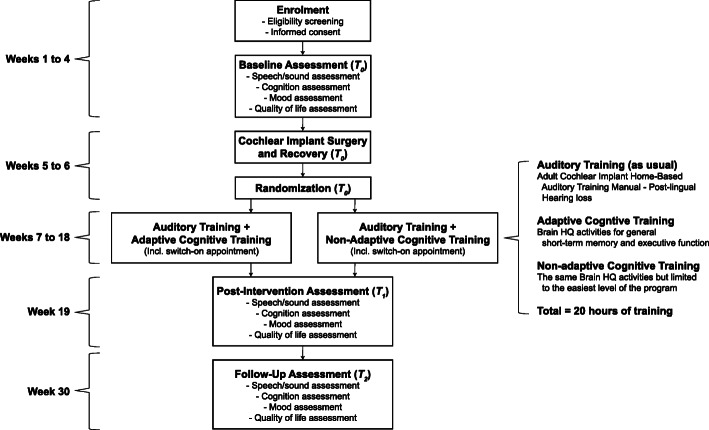
Flow diagram of testing procedure

A single-blind, head-to-head, randomised controlled trial (RCT) design will be used to determine whether auditory training combined with adaptive CCT will elicit greater improvement in study outcomes in adult cochlear implant recipients, when compared to auditory training combined with non-adaptive (i.e. placebo) CCT (see Fig. 1). Following enrolment into the study, participants will complete a pre-intervention assessment (*T*_0_) of speech/sound perception, cognition, mood, and quality of life, followed by their cochlear implant surgery and randomisation to either the intervention group (auditory training + adaptive CCT) or the control group (auditory training + non-adaptive CCT). Participants will then complete the 12-week training intervention followed by a post-intervention assessment (*T*_1_) and 3-month follow-up assessment (*T*_2_) involving the same outcomes measured at pre-intervention. Previous cognitive training studies have used a training period of 4–8 weeks [17]. For the current study, a training intervention of 12 weeks has been chosen to ensure participants gain the full benefits of the training. A follow-up at 3 months has been deemed appropriate to ensure continued participant interest and prevent high attrition rates. The use of an active control group will ensure that participants are blind to whether they are receiving adaptive or non-adaptive training, which helps eliminate potential placebo effects of training. The RCT will be conducted at Ear Science Institute Australia, Perth, Western Australia. Ethics approval for the study was received from the University of Western Australia Human Research Ethics Committee (RA/4/20/5287), and the trial was registered with the Australian and New Zealand Clinical Trials Registry (ACTRN12619000609156).

### Participants

Post-lingually deafened adults who meet clinical criteria [18] for a unilateral cochlear implant will be recruited from the Ear Science Implant Clinic in Perth, Western Australia. All participants will provide informed consent prior to participating in the study, and all the assessments and training will be conducted according to the University of Western Australia Human Research Ethics Committee guidelines.

#### Inclusion criteria

To be included in this study, participants must meet the following inclusion criteria: (1) 18 years of age or older, (2) have bilateral symmetrical sensorineural hearing loss, (3) met clinical criteria for their first (unilateral) cochlear implant, (4) be a native English speaker, or have spoken English for the past 10 years or longer, and (5) have access to a home computer and internet.

#### Exclusion criteria

Participants will be excluded from the study if they (1) demonstrate a cognitive decline measured by the Hearing Impaired Montreal Cognitive Assessment (HI-MoCA) screening test as a total score ≤ 25/30 [19], (2) demonstrate a pre-lingual hearing impairment, (3) have previously received a cochlear implant, (4) have previous experience of or currently use cognitive training, and (5) are unable to use or have access to a personal computer connected to the internet.

### Outcome measures

The primary outcome measures will be cognitive functioning and speech/sound perception as described below. The secondary outcome measures will be mood and quality of life as described below. All outcome measures will be measured as discrete or continuous data.

#### Cognition

In accordance with the Cattell-Horn-Carrol-Miyake (CHC-M) taxonomy of cognitive outcomes [20], a battery of non-verbal neuropsychological tests will be used to measure broad ability domains of general short-term memory (Gsm) and executive function (EF). Six tests will be used to measure each narrow ability domain within the broad domains of Gsm and EF.

#### High-working memory

Within the broad domain of Gsm, the narrow ability of high-working memory (high-WM) will be measured using an adapted non-verbal version of the Letter Number Sequencing (LNS) test from the Wechsler Adult Intelligence Scale-IV [21]. In this adapted non-verbal version of the LNS test, participants will be presented with task instructions/item lists on a computer screen. Following the presentation of each list, participants will be required to recall the list using paper and pencil to record their answers. Individual letters and numbers within each list will appear on the computer screen for 1000 ms, and a command “RECORD YOUR ANSWER” will appear between each trial. Scores will range from 0 to 30 with a higher score indicative of greater high-WM [21]. This adapted version of the LNS test will include the same items and trials as the original test and scores will range from 0 to 30 with a higher score indicative of greater high-WM [21]. The LNS test is classified as a measure of high-WM within Gsm because completion of the task involves the simultaneous processing of multiple streams of information (i.e. letters and numbers) while also storing task instructions to successfully coordinate the correct recall order [20].

#### Low-working memory

Within the broad domain of Gsm, the narrow ability of low working memory (low-WM) will be measured using the Paired Associates Learning (PAL) test. The PAL test is a non-verbal subtest within the Cambridge Neuropsychological Test of Automated Batteries (CANTAB™) [22] and involves the initial presentation of six greyed-out boxes that are ‘opened’ in a randomised order on the screen. One or more of the boxes will contain a coloured pattern. Coloured patterns are then presented in the middle of the screen, one at a time, and participants will need to select which box the same pattern was originally located within (CANTAB™) [22]. Task difficulty increases as more patterns are included and therefore need to be remembered within each trial. Performance on the PAL test will be scored as the number of trials required to locate the pattern(s) correctly. The PAL test is classified as a measure of low-WM within Gsm because the processing and storage of a single-stream of visual information (i.e. an abstract pattern) while also remembering different spatial locations of the items according to their original location is required to successfully complete the task [20].

#### Short-term memory

Within the broad domain of Gsm, the narrow ability of short-term memory (STM) will be measured using the Delayed Matching to Sample (DMS) test. The DMS test is a non-verbal subtest within the CANTAB™ [22] and involves the presentation of an abstract visual pattern followed by an immediate presentation or a short delay (4 or 12 s) then the presentation of four similar patterns. Using a touch screen tablet, participants will be instructed to select one of the four patterns that match with the original pattern presented in the centre of the screen (CANTAB™) [22]. Performance on the DMS test will be scored as the number of correct responses. The DMS test is classified as a measure of STM within Gsm because the processing and storage of a single-stream of visual information (i.e. an abstract pattern) in conjunction with an immediate or short-term delayed recall is required to successfully complete the task [20].

#### Updating

Within the broad domain of EF, the narrow ability of updating will be measured using a spatial *N*-back task. Based upon original work by Jaeggi, Buschkuehl, Jonides, and Perrig [23], Brain Workshop (http://brainworkshop.sourceforge.net/) developed an open source adaption of the *N*-back task using varying stimuli and conditions. The spatial *N*-back task to be used in this study will be a non-verbal measure of updating ability and will involve the presentation of coloured squares within different locations of a 3 × 3 grid. Each square will appear for 500 ms followed by a 2500-ms delay before the next square appears in a different (or the same) location. Participants will be required to determine and respond if the current stimulus appeared in the same location as the stimulus that appear *N* items back in the series. Participants will complete 30 trials of a 2-back condition and performance will be scored as percentage of correct responses. The spatial *N*-back task is classified as a measure of updating within EF because successful completion requires participants to serially update items held in their memory, by simultaneously adding and discarding items with the presentation of each stimulus [20].

#### Shifting

Within the broad domain of EF, the narrow ability of shifting will be measured using an adapted non-verbal version of the Trail Making Test-BA (TMT-BA) [24]. Part A of the TMT-BA requires participants to connect a series of numbered circles in ascending order and as quickly as possible. Part B requires participants to connect a series of numbered *and* lettered circles in ascending order, switching between letters and numbers (e.g. A–1–B–2), and as quickly as possible. Performance on parts A and B will be scored as the time taken to complete each task in seconds. The TMT-BA outcome score will then be computed as the time taken to complete part B minus the time taken to complete part A, with a larger time difference indicative of greater cost of switching between trials. The TMT-BA is classified as a measure of shifting within EF because successful completion of part B requires participants to hold two competing rules in mind while also alternating between each rule [20].

#### Inhibition

Within the broad domain of EF, the narrow ability of inhibition will be measured using the Multitasking Test (MTT) (CANTAB™) [22]. The MTT is a non-verbal subtest within the CANTAB™ and involves the presentation of an arrow on either the left or right side of the screen and the arrow can also point either left or right. Using two response pads located on the left and right sides of the bottom of the screen, participants will be asked (in some trials) to indicate whether the arrow was pointing left or right or whether the arrow was appearing on the left or right side of the screen. Some trials will display congruent stimuli (e.g. an arrow pointing left and on the left side of the screen) and some trials will display incongruent stimuli (e.g. an arrow pointing left but on the right side of the screen) (CANTAB™) [22]. The MTT is classified as a measure of inhibition within EF because successful completion of the task requires an active and deliberate overriding of an automatic response (i.e. to the direction of the arrow) to complete the task in accordance with incongruent information that may affect performance [20].

#### Sound and speech perception

A standardised pure-tone audiometric assessment (Equinox 2.0 clinical audiometer) will be used to assess participant pure-tone average (PTA) hearing thresholds across octave frequencies between 0.25 and 8 kHz. In accordance with clinical guidelines for cochlear implantation [18], speech perception will be assessed using an open-set list of 25 monosyllabic words (consonant-vowel-consonant [CVC] words [25]) scored as the percentage of phonemes and words correct. Speech perception will also be assessed using the open-set City University of New York (CUNY) sentences [26] and scored as percentage correct. Pre-implantation speech perception will be delivered via optimised hearing aids in quiet, with words and sentences assessed up to 65 dB through unilateral and bilateral hearing aids. Post-implantation speech perception assessments will be conducted using the speech processor.

#### Mood

The Depression, Anxiety, and Stress Scale (DASS-21) will be used to assess presence of depression, anxiety, or stress that may be associated with hearing loss and cognition [27]. Participants will be asked to report the degree to which they experience a range of psychological symptoms over the past week, using a 4-point Likert scale ranging from 0 (*did not apply to me at all*) to 3 (*applied to me very much*). For each dimension, scores may range from 0 to 21, with higher scores indicating greater experience of depression, anxiety or stress. The DASS-21 will be completed during pre-intervention, post-intervention, and 3-month follow-up assessments.

#### Quality of life

The Nijmegen Cochlear Implant Questionnaire (NCIQ) will be used to assess quality of life at pre-intervention, post-intervention, and follow-up assessments [28]. The NCIQ includes 60 items which ask participants to report their daily experiences associated with their hearing and the impact of their hearing on their life. Participant responses are measured on a Likert scale ranging between “Never” and “Always” and participant scores can range from 0 to 100, with a higher score indicative of worse quality of life [28].

### Screening/demographic measures

#### General cognition

The Hearing-Impaired Montreal Cognitive Assessment (HI-MoCA) will be used to assess general cognitive function and screen/exclude participants with dementia at pre-intervention assessments [19]. The HI-MoCA is based upon the original MoCA and has been developed to measure general cognitive function among adults with hearing loss, by converting the measure into a power-point slide show that is not dependent upon an individual’s hearing ability. Scores range from 0 to 30, with a score of 25 or less indicative of cognitive decline.

#### Cognitive reserve

The Cognitive Reserve Index questionnaire (CRIq) will be used to assess cognitive reserve at pre-intervention assessments [29]. The CRIq records demographic information, years of education, working activity, and leisure time experiences from adult life to compute a summary index of an individual’s level of cognitive reserve. Summary index scores range from ≤ 70 (low cognitive reserve) to ≥ 130 (high cognitive reserve).

#### Treatment expectations

The Stanford Expectations of Treatment Scale (SETS) will be used to assess participants’ baseline expectations of their anticipated response to the treatment (i.e. cochlear implant and auditory/cognitive rehabilitation) [30]. The SETS include 10 items, with six items relating to a participants’ potential positive or negative belief of their future response to a treatment. An example item includes, “My condition will be completely resolved after treatment” and responses range from “Strongly Disagree” to “Strongly Agree”. Treatment expectations will only be recorded at baseline and performance on the SETS will be analysed in relation to study outcomes and may be controlled for in outcome results.

#### Demographic characteristics

Participants will be asked to complete a short demographic questionnaire asking their age, gender, marital and occupational status, current leisure activities, and health/medical history.

### Test procedure

Participants who meet the clinical criteria for a cochlear implant will be recruited into the study. Participants will complete audiometric testing and speech/sound perception assessment within the 12 months preceding surgery and complete the pre-intervention cognitive, mood, and quality of life assessment within 1 week prior to implant. Participants will have the option to complete the pre-intervention assessment in their home or at the Ear Science Institute Australia at a time that is most convenient to them. Following cochlear implant surgery and recovery, participants will attend their ‘switch-on’ appointment where they will be provided with the standardised auditory training material by their implant audiologist. Following ‘switch-on’ appointments, participants will be randomised to either the adaptive or non-adaptive CCT group and the lead researcher will schedule a time within the same week to visit participant homes to set up the CCT equipment. As the CCT platform, Brain HQ will be delivered via the internet to participant’s home computers and the lead researcher will spend time explaining and demonstrating how to use the platform. Participant adherence to the training will be automatically monitored by the program and remotely by the lead researcher. Participants will be sent regular text messages during the intervention as polite reminders to complete their CCT sessions. Following completion of the training, participants will complete audiometric testing and a post-intervention assessment of their speech/sound perception, cognition, mood, and quality of life. The same outcomes will also be measured at the 3-month follow-up assessment to provide evidence of long-term benefits of CCT.

### Randomisation and blinding

The first participant recruited to the trial will be randomised to either the adaptive or non-adaptive CCT group. The following participants will then be allocated to either group using minimization [31]. Participant group allocation details will be documented and stored securely by the chief investigator in a password-protected file. As a single-blind trial, participants will be blind to group allocation and it will not be possible for participants to tell from the online CCT interface which group they have been assigned to.

### Interventions

#### Auditory training

All participants will complete auditory training following the Adult Cochlear Implant Home-Based Auditory Training Manual – Post-lingual Hearing Loss [14]. Published by Cochlear®, the auditory training manual provides a series of activities of increasing difficulty. Listening activities range from ‘Module 1’ which involves daily practice of listening and identifying environmental sounds (e.g. kettle boiling, phone ringing) to ‘Module 16’ which involves partnered conversations where participants are required to repeat what is heard and most importantly, what is understood, during a conversation [14]. The auditory training manual also includes instructions for completing computer-based activities via the Angel Sound™ program [14]. Angel Sound™ is an adaptive auditory training program designed for CIRs and involves similar listening activities to those included in the training manual. Participants will be encouraged to use Angel Sound™ in conjunction with the training manual activities. Participants will be instructed to remove their contralateral hearing aid to ensure reliance upon and increased use of the cochlear implant during training.

#### Adaptive computerised cognitive training

The Brain HQ (Posit Science™) program will be used for adaptive CCT. Brain HQ is a commercially available auditory-cognitive training program and findings from our recent meta-analysis [17] suggest that Brain HQ (previously known as Brain Fitness) may improve cognition in adults with hearing loss. There is also high quality (i.e. Level 1) evidence in support of the efficacy of Brain HQ for improving cognition in older adults [32]. To determine whether CCT provides an additive therapeutic effect on study outcomes beyond the standardised auditory training regime, participants will only complete *visual* CCT activities that target cognitive domains (i.e. Gsm and EF) involved in the successful processing of sound and speech. CCT will therefore place no demand upon a participant’s hearing ability and will not interfere with the standardised post-implant auditory training rehabilitation protocol as recommended by each participant’s implant audiologist. In accordance with the CHC-M taxonomy of cognitive outcomes (Webb et al., 2018), Brain HQ CCT will involve the following activities designed to train each narrow cognitive ability within their corresponding broad domain: (1) ‘Juggle Factor’ will train high-WM within Gsm, (2) ‘Mind’s Eye’ will train low-WM within Gsm, (3) ‘Scene Crasher’ will train STM within Gsm, (4) ‘Card Shark’ will train updating within EF, (5) ‘Mind Bender’ will train shifting within EF, and (6) ‘Freeze Frame’ will train inhibition within EF. Participants will begin the training protocol on the least challenging level and the program will increase or decrease (i.e. adapt) task difficulty depending on participant progress during training. Participants will complete 20 min of CCT, 5 days a week for 12 weeks, totalling 20 h of CCT.

#### Non-adaptive computerised cognitive training

The same training protocol and Brain HQ exercises will be completed by participants in the non-adaptive CCT group to ensure intervention parameters (i.e. stimuli, length of training) are equal across groups. However, training exercises will be limited to the least challenging level (i.e. non-adaptive) to ensure no therapeutic benefit is experienced during training.

### Data management

All participant data will be securely stored in locked cabinets and password protected files on the chief investigator’s computer at Ear Science Institute Australia. Identifiable information of participants will be kept separate from participant results on outcome measures, and only anonymous results will be analysed and reported in research outputs (i.e. journal articles, conference presentations) pertaining to this research.

### Data analysis and statistical methods

SPSS (version 26) will be used to calculate descriptive statistics for demographic data and outcome test results at pre-intervention, post-intervention, and 3-month follow-up assessments. Generalised linear mixed models (GLMMs) will be used to analyse outcome variables. Each GLMM will be assessed for statistically significant Group x Time interaction effects, main effects of Time (per group), and pairwise contrasts. Age effects on cognitive training will be considered in the statistical analysis and results will be discussed accordingly.

Following a wave of recent criticism drawing attention to the limitations of the frequentist approach (i.e. *p* values and alpha levels) of statistical analysis, there has been increasing recommendations for researchers to report Bayes factors to support the interpretation of their findings [33]. Within the frequentist approach, it is most frequently misunderstood that a statistically significant *p* value (i.e. *p* ≤ .05) provides evidence in support of an alternative hypothesis, whereas a statistically significant *p* value can only provide evidence to disprove the null hypothesis and cannot suggest whether the observed data supports an alternative hypothesis [33]. Compared to the frequentist approach, however, the Bayesian framework allows researchers to quantify whether changes in their observed data favour the null hypothesis or their alternative hypothesis by considering prior odds (i.e. prior evidence of similar effects). It is therefore important that researchers report Bayes Factors alongside frequentist statistics to provide a more informative interpretation of their findings. From the GLMM results, Bayes Factors will be approximated using the Bayesian Information Criterion (BIC) from each model (i.e. H0 and H1) and reported to provide evidence of whether auditory training combined with adaptive CCT improves study outcomes to a greater extent, when compared to auditory training combined with non-adaptive (i.e. placebo) CCT.

### Power analysis and sample size

Evidence from our recent meta-analysis of cognitive training for adults with hearing loss reported beneficial effects ranging from small to large [17], with contributing studies including 10 to 67 participants. Using G*Power (34), an a priori power analysis was conducted to determine the required sample size when comparing two intervention groups across three measurement intervals (i.e. pre-intervention, post-intervention, and 3-month follow-up) for any of the primary outcome measures (speech/sound perception or cognition). To detect a medium effect (*f* ^2^ = .25) at an alpha of .05 and power of .90, 36 participants will need to be recruited. To account for potential 20% attrition, 44 participants (i.e. 22 per group) will be targeted for recruitment.

## Discussion

Adults with severe to profound hearing loss are at increased risk of experiencing cognitive decline, depression, and poor quality of life. Cochlear implantation is a reliable and safe procedure for restoring sound and speech perception in adults with severe to profound hearing loss, but the potential benefits of a cochlear implant for improving cognition, mood and quality of life are less clear.

Several studies have investigated the effect of cochlear implantation on the cognitive functioning of adults without specific training, and have found an improvement in cognitive functioning at follow-up [34, 35]. In a recent systematic review, five studies reported improved cognition following cochlear implantation. However, all these studies were limited by methodological biases that undermined reliable interpretation of results [8–13]. Regarding the impact of auditory training following cochlear implantation, only two of the studies in the systematic review reported that participants completed post-operative auditory rehabilitation [8, 12]. This is despite clinical guidelines recommending auditory rehabilitation for successful use and adjustment to a new cochlear implant [14]. It is therefore not clear whether cochlear implantation or cochlear implantation combined with post-operative auditory rehabilitation has the potential to improve cognition in adults following surgery. Regarding the impact of cognitive training following cochlear implantation, there has been remarkably little research in this area. However, the beneficial effects of cognitive training for healthy older adults appear to be well supported [15, 31, 36].

To determine whether auditory training combined with CCT improves cognition in cochlear implant recipients, we will implement a training paradigm that elicits improvement in on-task (trained) activities and off-task (untrained) outcome measures. The multi-domain CCT that we propose in this manuscript involves training multiple cognitive domains simultaneously (e.g. attention, short-term/working memory, executive function) and provides greater therapeutic benefit by targeting a combination of cognitive skills that likely overlap and are used in combination, in complex real-world situations [35, 36], since frontal cognitive resources (e.g. attention and working memory) are used during successful processing and interpretation of speech/sound [37] and meta-analytic evidence indications that adults with hearing loss primarily demonstrate deficits in attention, short-term/working memory, and executive function [38]. Combining multi-domain CCT with existing auditory training may lead to significant on-task and off-task improvement in cognition among cochlear implant recipients.

### Trial status

Participant recruitment has not been commenced. Anticipated date of the first participant recruitment 3 November 2021. Anticipated participant recruitment completion date 31 December 2023.

## Data Availability

This is a protocol for the clinical trial; data collection has not been commenced yet. Once the data collection is completed, data will be presented in subsequent manuscripts.

## Abbreviations

CI: Cochlear implants
RCT: Randomised controlled trial
CCT: Computerised cognitive training
WM: Working memory
LNS: Letter Number Sequencing
GLMM: Generalised linear mixed model
STM: Short-term memory
Gsm: General short-term memory
EF: Executive function
NCIQ: Nijmegen Cochlear Implant Questionnaire
BIC: Bayesian Information Criterion
CRIq: Cognitive Reserve Index questionnaire
CHC-M: Cattell-Horn-Carrol-Miyake
